# Lung protection during non-invasive synchronized assist versus volume control in rabbits

**DOI:** 10.1186/cc13706

**Published:** 2014-01-23

**Authors:** Lucia Mirabella, Giacomo Grasselli, Jack J Haitsma, Haibo Zhang, Arthur S Slutsky, Christer Sinderby, Jennifer Beck

**Affiliations:** Department of Anesthesiology, University of Foggia, viale Pinto 1, 71122 Foggia, FG Italy; Department of Health Sciences, University of Milano Bicocca, via Cadore 48, 20900 Monza, MB Italy; Department of Anesthesiology and Critical Care, Ospedale San Gerardo, via Pergolesi 33, 20900 Monza, MB Italy; Keenan Research Centre for Biomedical Science of St. Michael’s Hospital, room 611, 30 Bond Street, Toronto, Ontario M5B1W8 Canada; Department of Critical Care, St. Michael’s Hospital, 30 Bond Street, Toronto, Ontario M5B1W8 Canada; Department of Medicine and Interdepartmental, Division of Critical Care Medicine, University of Toronto, Toronto, Canada; Department of Pediatrics, University of Toronto, Toronto, Canada

## Abstract

**Introduction:**

Experimental work provides insight into potential lung protective strategies. The objective of this study was to evaluate markers of ventilator-induced lung injury after two different ventilation approaches: (1) a “conventional” lung-protective strategy (volume control (VC) with low tidal volume, positive end-expiratory pressure (PEEP) and paralysis), (2) a physiological approach with spontaneous breathing, permitting synchrony, variability and a liberated airway. For this, we used non-invasive Neurally Adjusted Ventilatory Assist (NIV-NAVA), with the hypothesis that liberation of upper airways and the ventilator’s integration with lung protective reflexes would be equally lung protective.

**Methods:**

In this controlled and randomized in vivo laboratory study, 25 adult White New Zealand rabbits were studied, including five non-ventilated control animals. The twenty animals with aspiration-induced lung injury were randomized to ventilation with either VC (6 mL/kg, PEEP 5 cm H2O, and paralysis) or NIV-NAVA for six hours (PEEP = zero because of leaks). Markers of lung function, lung injury, vital signs and ventilator parameters were assessed.

**Results:**

At the end of six hours of ventilation (n = 20), there were no significant differences between VC and NIV-NAVA for vital signs, PaO2/FiO2 ratio, lung wet-to-dry ratio and broncho-alveolar Interleukin 8 (Il-8). Plasma IL-8 was higher in VC (*P* <0.05). Lung injury score was lower for NIV-NAVA (*P* = 0.03). Dynamic lung compliance recovered after six hours in NIV-NAVA but not in VC (*P* <0.05). During VC, peak pressures increased from 9.2 ± 2.4 cm H2O (hour 1) to 12.3 ± 12.3 cm H2O (hour 6) (*P* <0.05). During NIV-NAVA, the tracheal end-expiratory pressure was similar to the end-expiratory pressure during VC. Two animals regurgitated during NIV-NAVA, without clinical consequences, and survived the protocol.

**Conclusions:**

In experimental acute lung injury, NIV-NAVA is as lung-protective as VC 6 ml/kg with PEEP.

**Electronic supplementary material:**

The online version of this article (doi:10.1186/cc13706) contains supplementary material, which is available to authorized users.

## Introduction

Ventilator-induced lung injury (VILI) is a topic of utmost importance in both the adult and infant populations. From animal studies, it is evident that in order to minimize VILI, lung over-distension should be avoided and positive end-expiratory pressure (PEEP) should be used to keep the lung from de-recruiting [1]. VILI studies in animals generally use the same model [2–5]: experimental lung injury is induced and then animals are randomized to different strategies and/or modes. The protective ventilation strategy commonly used is a controlled mode of low tidal volume (Vt) (usually 6 ml/Kg) with a fixed PEEP, applied with intubation and sedation, with or without neuromuscular paralysis.

Recent experimental studies suggest that spontaneous breathing [6], variability of breathing pattern [7], and proportionality [3, 8] can aid in the attenuation of VILI in intubated animals with mild early experimental lung injury (partial arterial pressure of oxygen (PaO2) to inspired oxygen fraction (FIO2) ratio (P/F) approximately 100 to 150). The work of Brander *et al*. in intubated rabbits with hydrochloric acid (HCl)-lung injury demonstrated that neurally adjusted ventilatory assist (NAVA) is at least as protective against VILI as the conventional protective ventilation strategy of volume control (VC) [3].

Interestingly, no studies have examined the use of non-invasive ventilation (NIV) in reducing VILI. Such an approach could provide even greater lung protection by allowing the naturally occurring regulation of end-expiratory lung volume [9] and prevention of atelectasis, as well as avoiding the complications associated with having an endotracheal tube in place (for example, ineffective cough, colonization of the tube, and tracheal and glottal injury).

Based on the above, we compared VILI with two ventilation approaches: (1) the conventional lung protective strategy where Vt and PEEP are controlled (6 ml/Kg and 5 cm H_2_O) and neuromuscular paralysis is used, or (2) a physiological approach with spontaneous breathing, variability and a liberated airway (without an imposed Vt and without PEEP). We used NIV-NAVA for the latter group, knowing that synchronous ventilation can be provided in the presence of large leaks [10], both in terms of timing and proportionality. We hypothesized that NIV-NAVA would be equally protective as the protective strategy in terms of VILI, because of: (i) the freedom to choose the breathing pattern, (ii) liberation of the upper airways, and (iii) integration of the ventilator with lung protective reflexes. Some of the results of this study have been previously reported in the form of an abstract [11].

## Materials and methods

The study was approved by St Michael’s Hospital Animal Care and Use Committee. Care and handling of the animals was in accordance with the Canadian Council on Animal Care.

### Animals

**Figure 1:**
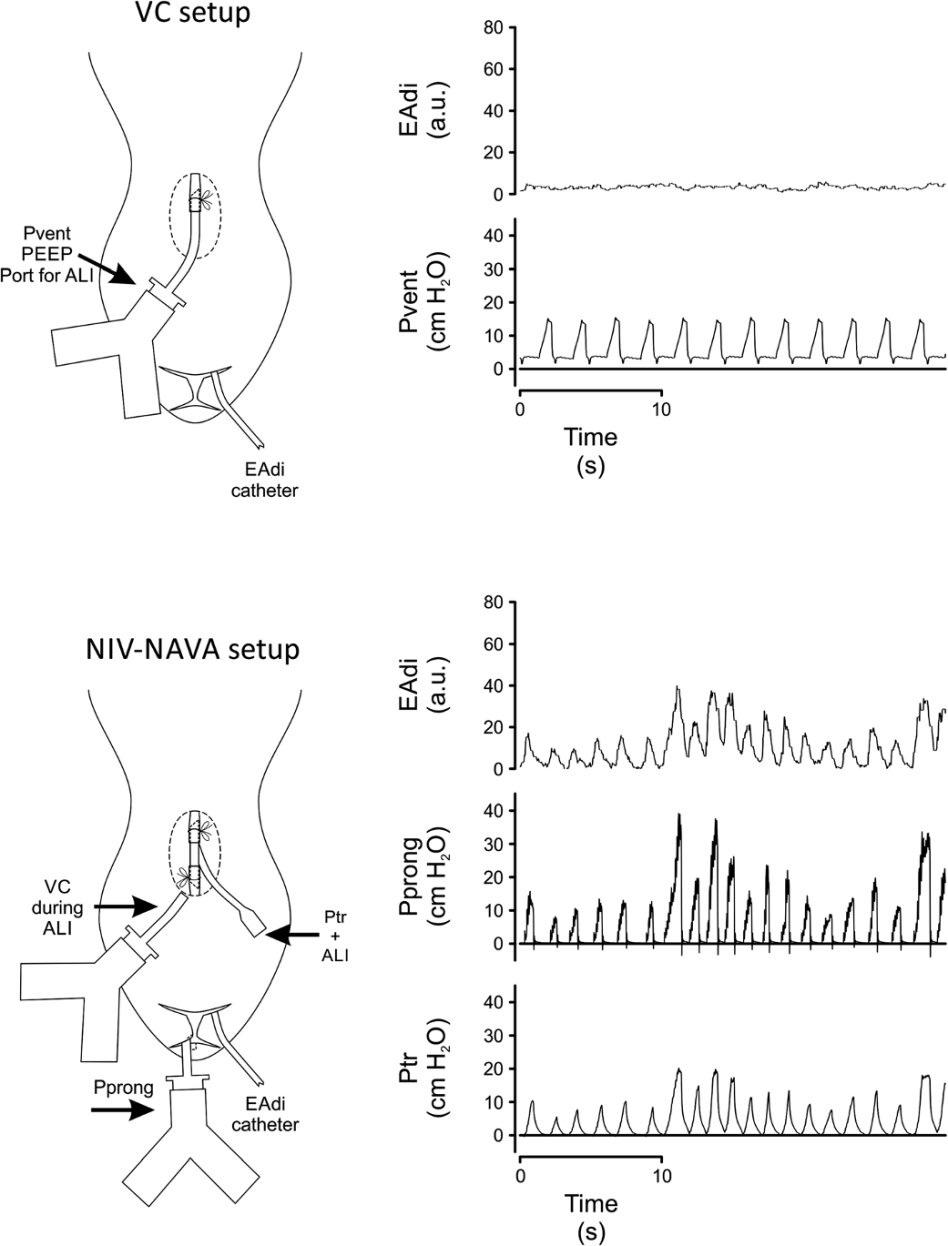
**Experimental set up and experimental recordings.** Left panels show experimental set-up and physical placement of interfaces and right panels show experimental recordings for each mode. Volume control (VC, top) and non-invasive ventilation-neurally adjusted ventilatory assist (NIV-NAVA, bottom). VC setup: ventilator was connected to the endotracheal tube at the tracheostomy to measure ventilator pressure (Pvent). NIV-NAVA setup: Ventilator was connected to the nasal prong (Pprong), where ventilator-delivered pressure was measured. Tracheal pressure (Ptr) was also measured via a side-port at the tracheotomy. PEEP, positive end-expiratory pressure; EAdi, electrical activity of the diaphragm.

Twenty five rabbits (3.1 ± 0.4 Kg) were studied (n = 10 in volume control, n = 10 in NIV-NAVA, n = 5 non-ventilated and non-injured controls). As previously described [3, 10, 12, 13], the ventilated animals received continuous infusion of ketamine hydrochloride (40 mg/Kg/h), xylazine (4 mg/Kg/h) and lactated Ringer’s solution (5 mL/Kg/h). Blood pressure, oxygen saturation, body temperature, and heart rate were monitored. An endotracheal tube was placed in the trachea but below the larynx (Figure 1). In addition, in the NIV-NAVA arm, a single nasal prong was placed into one nostril [10].

Pressure measurements are described in Figure 1. A Servo300 ventilator (Maquet, Sweden) was used and was connected via tubing to the trachea in VC (Pvent), or to the nasal prong in NIV-NAVA (Pprong). Electrical activity of the diaphragm (EAdi) was measured as previously described [3].

### Protocol

**Figure 2:**
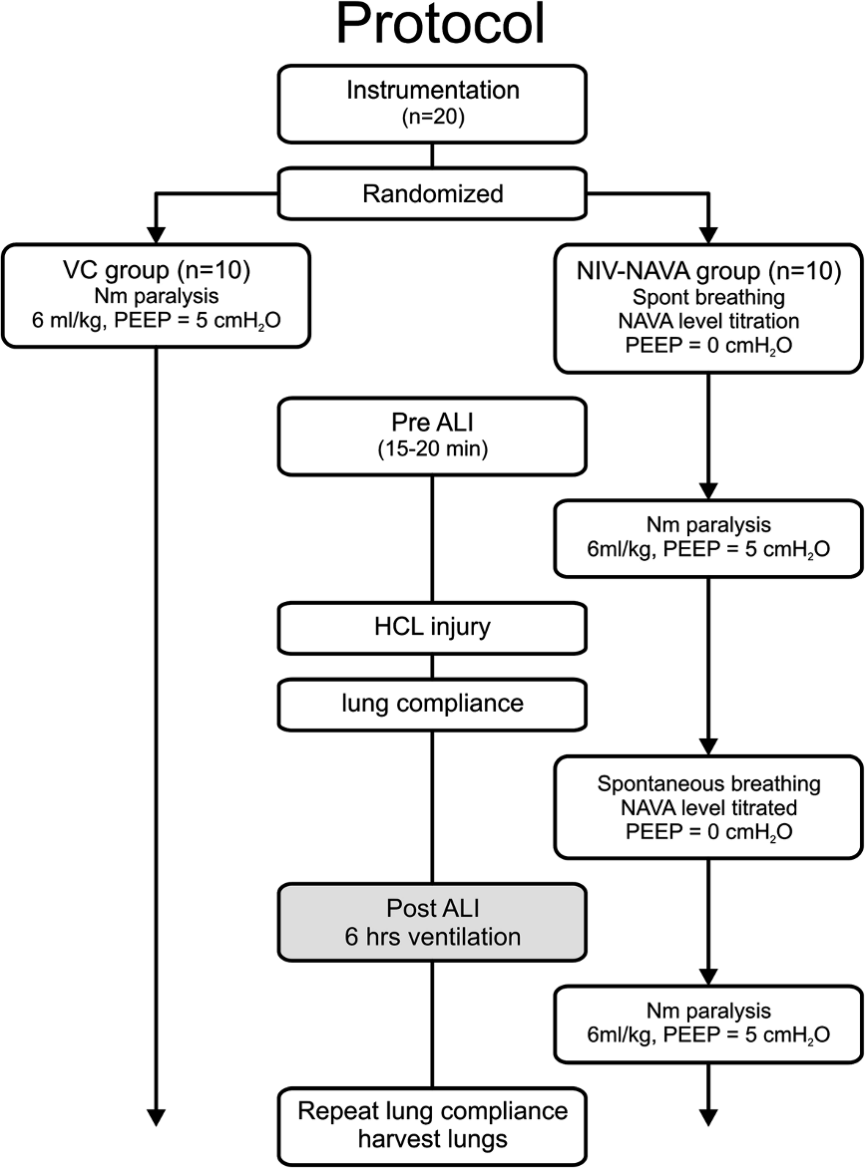
**Experimental protocol.** Animals were randomized to be ventilated with either volume control mode (with neuromuscular paralysis, VC group, left) or with non-invasive ventilation with neurally adjusted ventilatory assist (NIV-NAVA) and spontaneous breathing (NIV-NAVA group, right). Animals were briefly ventilated before an acid-induced acute lung injury (Pre-ALI) and for 6 hours (Post-ALI). In both arms, dynamic lung compliance was measured Pre-ALI, Post-ALI, and after 6 hours. PEEP, positive end-expiratory pressure.

Measurements were made before acute lung injury (Pre-ALI) and 5 minutes after (Post-ALI), and for 6 hours in either VC (paralyzed with 6 ml/Kg, PEEP 5 cm H_2_O) or NIV-NAVA (spontaneous breathing) (Figure 2). The method for NAVA has been previously described in detail [13] (see Additional file 1). The NAVA level was titrated [13–16] to allow determination of (i) maximal diaphragm activation during zero assist, and (ii) an adequate NAVA level. During NIV-NAVA, no effective PEEP could be applied because of the leak and because the prototype being used (Servo300) did not have a dedicated NIV mode with leak compensation.

During VC mode, the initial setting for PEEP on the ventilator was 5 cm H2O, but if necessary for hemodynamic reasons, PEEP was lowered in 1 cm H2O steps if mean arterial blood pressure decreased below 50 mmHg [3]. Respiratory rate was adjusted (if necessary) based on the arterial blood gases in the previous hour.

### Acute lung injury

After the Pre-ALI phase, both groups had induction of ALI by intratracheal instillation of HCl (pH 1.5) (1.5 mL/Kg) [3, 10], while receiving neuromuscular paralysis and controlled ventilation (Vt 6 mL/Kg; PEEP 5 cm H2O; FiO_2_ 50%). Total respiratory dynamic compliance (Cdyn, mL/cm H2O) was measured during paralysis Pre-ALI and Post-ALI using the formula: Vt/(Ppeak-PEEP). Static compliance was measured as Vt/(Pplat-PEEP).

Animals were then ventilated for six hours with either VC or NIV-NAVA, and arterial blood samples were taken hourly. All other measurements were recorded for the last 20 minutes of each hour. At the end of the protocol, Cdyn was re-measured for both modes.

### Lung injury markers

After sacrifice, the heart-lung block was removed, the lungs were inflated and the main left-side bronchus and the right lower-lobe bronchus were tightly occluded. Saline was instilled and filled the non-occluded right lung for bronchoalveolar lavage (BAL), and was then aspirated and immediately centrifuged and stored. The right lower lobe was used to measure lung wet-to-dry ratio. The remaining right lung was used for tissue IL-8. Rabbit IL-8 was measured in BAL fluid and plasma (at 3 hours and 6 hours) using a human IL-8 ELISA kit (Biosource International, Camarillo, CA, USA) [3]. See Additional file 1 for the rationale of using IL-8, as well as references [17, 18].

### Histology

Axial lung slices (thickness 0.5 cm) were made of the fixated lung 0.5 cm caudal of the lung hilus. The lung slice was subsequently divided into three parts representing dependent, intermediate and non-dependent lung areas with a minimal margin of 3 mm in between the areas, as well as with the lung edge. In our supine-positioned animal, the dependent lung area was defined as the most dorsal lung section, non-dependent corresponded with the ventral lung area, and intermediate the area in between the dependent and non-dependent lung areas [19]. The specimens were embedded in paraffin, sectioned and stained with hematoxylin and eosin. The analyzing pathologist, blinded to study group, scored the samples for lung injury (see Additional file 1) [20].

### Ventilatory variables

Analysis of EAdi and respiratory variables is described in Additional file 1 and in references [13–16, 21]. Briefly, the EAdi waveform was quantified by its phasic component (that is, the inspiratory change in amplitude from baseline to peak) and the minimum EAdi, or tonic EAdi, during exhalation. The phasic EAdi was related to the maximum EAdi at zero assist during the NAVA titration, to obtain a relative inspiratory EAdi. The pressure waveforms were also quantified by their inspiratory (delta) change during inspiration, and the mean expiratory pressure.

### Statistics

Data are presented as mean ± SD or median (quartiles), depending on whether or not the data were normally distributed. Two-way repeated measures analysis of variance (RM-ANOVA) was used to compare variables over time and between modes. The Student Newman-Keuls test was used for post hoc analysis: the *t*-test was used to compare the IL-8 concentration in blood samples and BAL, and lung injury score (LIS). A significant difference was defined as *P* <0.05. SigmaStat was used for statistical analyses (Sigmastat, Jandel Scientific, San Jose, CA, USA).

## Results

All twenty animals survived the protocol. In the NIV-NAVA arm, the average time for EAdi recovery after paralysis was 21 ± 7 minutes. Two animals in the NIV-NAVA arm regurgitated half way through the 6-hour protocol. These animals did not demonstrate any clinical signs of aspiration (no changes in breathing pattern or P/F ratio), and were not excluded from the analysis.

### HCl-induced lung injury

**Figure 3:**
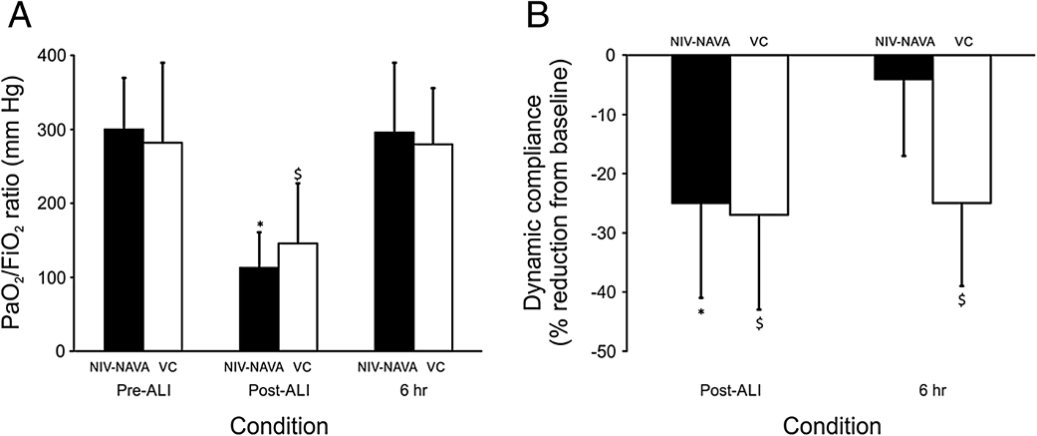
**Partial arterial pressure of oxygen/inspired oxygen fraction (PaO**_**2**_ **/FIO**_**2**_ **) ratio and dynamic lung compliance after lung injury and after 6 hours of ventilation. (A)** PaO_2_/FIO_2_ ratio before acute lung injury (Pre-ALI), Post-ALI, and 6 hours after ALI with non-invasive ventilation with neurally adjusted ventilatory assist (NIV-NAVA, black bars) and volume control (VC, white bars). **(B)** Reduction in dynamic compliance Post-ALI, and after 6 hours of ventilation with NIV-NAVA (black bars) and VC (white bars) expressed as percent reduction from Pre-ALI. Note that dynamic compliance recovered in the NIV-NAVA group, but not the VC group. Values are mean ± SD. *NIV-NAVA, significantly different from Pre-ALI. ^**$**^VC, significantly different from Pre-ALI.

After ALI, the mean P/F ratio was reduced from 300 ± 70 mmHg to 113 ± 48 mmHg for the NIV-NAVA arm (*P* <0.001), and from 282 ± 108 to 146 ± 81 for the VC arm (*P* = 0.002), and was not different between the two groups (Figure 3A). Total respiratory dynamic compliance was reduced significantly after ALI in both groups (by 25 ± 16% in the NIV-NAVA group, and 27 ± 17% in the VC group, *P* <0.001), and were not different from each other (Figure 3B). Absolute values for dynamic compliance are presented in Table 1.

**Table 1:** **Timeline of vital signs and ventilator parameters**

	Mode	Pre-ALI	Post-ALI	1 hour	2 hours	3 hours	4 hours	5 hours	6 hours	***P*** -value (time)	***P*** -value (mode)
**Mean BP, mmHg**	NIV-NAVA	83^$^	70	79^$^	70	68	61	61	61	NS	*P* <0.05 at Pre-ALI and 1 h
		(77,88)	(50,87)	(73,86)	(66,80)	(62,77)	(58,75)	(54,70)	(50,68)		
	VC	67	72	72	73	65	64	56	66	NS	
		(64,80)	(64,80)	(65,77)	(67,77)	(57,68)	(56,67)	(52,67)	(53,70)		
**Heart rate, bpm**	NIV-NAVA	156	202 ^#^	172	171	174	179	176	178	*P* <0.05	NS
		(152,169)	(190,212)	(153,181)	(163,193)	(170,179)	(166,183)	(160,181)	(161,190)	Post -LI	
	VC	150	193 ^#^	162	172	176	179	176	174	*P* <0.05	
		(142,195)	(178,202)	(148,183)	(151,185)	(166,198)	(166,183)	(160,181)	(150,196)	Post-ALI	
**Body temperature, °C**	NIV-NAVA	38.9	39.1	39.2	38.9	39.0	38.9	39.0	38.9	NS	NS
		(38.7,39.1)	(38.9,39.2)	(38.9,39.5)	(38.8,39.6)	(38.8,39.4)	(38.6,39.3)	(38.6,39.3)	(38.7,39.2)		
	VC	39.0	39.1	39.0	39.2	39.2	39.2	39.0	39.1	NS	
		(38.5,39.3)	(38.5,39.2)	(38.8,39.2)	(38.9,39.3)	(39.0,39.6)	(38.8,39.4)	(38.8,39.1)	(38.9-39.3)		
**SAO2,%**	NIV-NAVA	99.1	91.1^# $^	98	97.3	98.7	98.4	98.8	98.6	*P* <0.05	*P* <0.05 at Post-ALI
		(98.3,99.3)	(75.3,97)	(95.7,99)	(93.7,98.9)	(96.5)	(97.1,98.8)	(97,99)	(97.4,99.1)		
	VC	98.5	92.1	98.8	98.7	98.6	98.9	98.8	99.1	NS	
		(97.4,99.1)	(77.2,97)	(98,99.4)	(98.5,99)	(97.4,99)	(98.2,99.3)	(98.2,99.1)	(98.1,99.1)		
**PaCO2, mmHg**	NIV-NAVA	55.2	73.7 ^#$^	54.5	50.6	45.8	51.6	50.7	53.8	*P* <0.05	*P* <0.05 Post -LI
		(51.7,59.9)	(62.8,77.9)	(42.8,63.6)	(47.3,59.5)	(40.8,63)	(38.8,56.1)	(38.9,56.9)	(32.8,61)		
	VC	56.4	56.6	50.15	49	50.7	46.2	47.1	47.3	NS	
		(47.2,57.9)	(45.3,63.2)	(45.5,55.7)	(42,55)	(40,55.6)	(39.3,48.8)	(42,52.1)	(38.6,60.8)		
**Ventilation rate per minute**	NIV-NAVA	30	47^#$^	36^$^	36^$^	32	29	37^$^	37^$^	*P* <0.05	*P* <0.05 Post-ALI
		(± 7)	(±15)	(±8)	(±9)	(±7)	(±6)	(±24)	(±25)		
	VC	28	26	26	26	27	27	28	28	NS	
		(±9)	(±6)	(±4)	(±4)	(±3)	(±3)	(±2)	(±2)		
**Cdyn, mL/cm H2O**	NIV-NAVA	1.73^$^	0.94^#^	-	-	-	-	-	1.18*	*P* <0.01	*P* <0.05 Pre-ALI
		(±0.25)	(±0.25)						(±0.14)		
	VC	2.43	1.26^#^	-	-	-	-	-	1.30^#^	*P* <0.05	
		(±0.76)	(0.33)						(±0.29)	vs. Pre-ALI	
**Cstat, mL/cm H2O**	VC	2.5	2.1^#^	2.3^#^	2.0^#$^	1.9^#$^	1.9^#$^	1.7^#$^	1.8^#$^	*P* < 0.001	-
		(2.5,2.8)	(1.7,2.3)	(2.1,2.7)	(1.9,2.4)	(1.5,2.3)	(1.5,2.3)	(1.5,2.0)	(1.5,2.1)	All vs. Pre-ALI and 1 h	
**Plateau pressure, cmH2O**	VC	8.4	11.9^#^	11.5^#^	11.9^#^	12.7^#^	12.3^#^	12.5^#^	12.9^#^	*P* <0.05	-
		(7.2,9.0)	(10.8,12.6)	(9.2,13.7)	(10.5,14.0)	(10.3,13.4)	(10.8,14.3)	(11.3,14.7)	(11.1,14.0)	All vs. Pre-ALI	
**Inspiratory flow, mL/s**	VC	19.2	21.2	20.9	21.3	21.6	21.7	23.2	23.6	NS	-
		(15.2,21.1)	(17.6,33.1)	(15.9,23.7)	(15.8,23.8)	(16.0,30.0)	(20.7,29.9)	(20.6,29.6)	(20.4,31.7)		

Median values (25th to75th percentile) unless otherwise indicated, by ‘ ± ’. BP, blood pressure; SAO2, saturation from arterial blood gas; Cdyn dynamic compliance; PaCO2, arterial carbon dioxide; NIV-NAVA, non-invasive NAVA; VC, volume control; ALI, acute lung injury. ^#^Within a mode, significantly different from prior to acute lung injury (Pre-ALI); vs., versus; NS, not significant. ^*^Within a mode, significantly different from Post-ALI. ^$^Between modes, significantly different at specific time point.

### Six-hour protocol

After 6 hours of ventilation, the P/F ratio recovered in both groups back to Pre-ALI values (Figure 3A). On the other hand, dynamic compliance recovered in the NIV-NAVA group, but did not in the VC animals (Figure 3B). Similarly, static compliance decreased consistently throughout the 6 hours of VC ventilation, and was significantly lower at hour 6 compared to hour 1 (Table 1).

**Figure 4:**
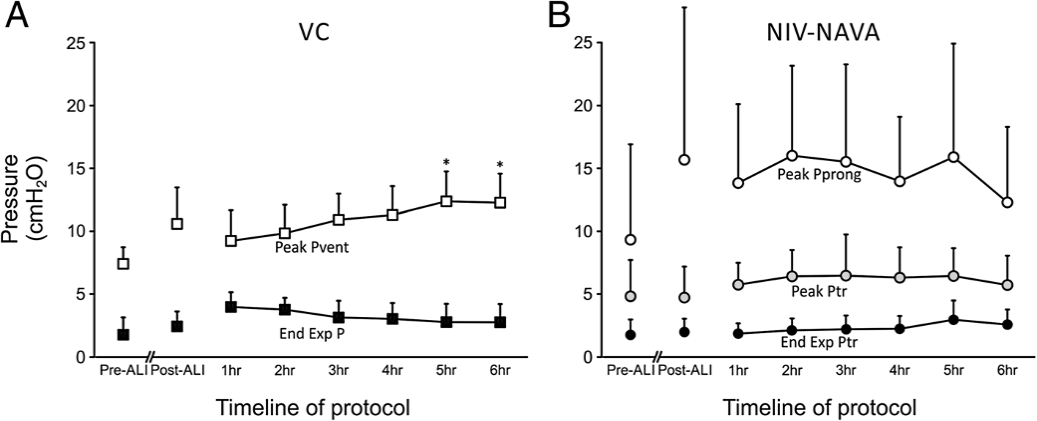
**Ventilator pressures during volume control (VC), and prong and tracheal pressure during non-invasive ventilation with neurally adjusted ventilator assist (NIV-NAVA). (A)** End-expiratory pressure (End Exp P, solid squares) and peak inspiratory pressure (above End Exp P, open squares) during VC in pre-acute lung injury (Pr-ALI) conditions, Post-ALI, and during 6 hours of ventilation. Peak inspiratory pressures increased during hours 5 and 6. **(B)** End-expiratory tracheal pressure (solid black circles), peak inspiratory tracheal pressure (gray solid circles), and peak prong pressure (open circles) during NIV-NAVA in Pre-ALI conditions, Post-ALI, and during 6 hours of ventilation. Values are mean ± SD. *VC, significantly different from 1 h. Ptr, tracheal pressure; Pvent; ventilator pressure.

In VC mode (Vt = 6 mL/Kg), there was a significant increase in peak ventilator pressure from 9.2 ± 2.4 cm H_2_O at hour 1 to 12.3 ± 12.3 cm H_2_O at hour 6 (*P* <0.05) (Figure 4A). Also, plateau pressure had a tendency to increase throughout the 6 hours (11.8 (10.8 to 12.6) to 12.9 (11.1 to 14.0) cm H_2_O) (Table 1). Inspiratory flow during VC mode was not significantly different throughout the protocol (Table 1).

During NIV-NAVA (spontaneous breathing), the peak delivered pressure measured at the nasal prong was not different between hour 1 (13.8 ± 6.3 cm H_2_O) and hour 6 (12.3 ± 6.0 cm H_2_O) (Figure 4B). Peak inspiratory Ptr during NIV-NAVA did not significantly change over time (4.8 ± 2.9 versus 5.7 ± 2.3 cm H_2_O). With the large leak, the pressure in the trachea was 42 ± 6.2% of the pressure measured at the nasal prong (range 40% to 46% during the 6 hours of NIV-NAVA). The evolution of the leak (or pressure transmission), is presented in Table 2 and was not significantly different over time.

**Table 2:** **Timeline of diaphragm electrical activity (EAdi) and pressure transmission to the trachea with non-invasive neurally adjusted ventilator assist (NIV-NAVA)**

	Pre-ALI	Post-ALI	1 hour	2 hours	3 hours	4 hours	5 hours	6 hours	***P*** -value (time)
**EAdi peak, a.u.**	22.1 (±10.6)	18.2 (±12.4)	15.6 (±4.8)	19.1 (±6.5)	18.0 (±7.3)	18.9 (±6.2)	21.1 (±9.9)	17.3 (±8.0)	NS
**EAdi minimum, a.u.**	5.2 (±1.7)	5.6 (±1.5)	4.5 (±1.8)	4.9 (±1.0)	4.8 (±1.2)	4.7 (±1.4)	5.6 (±3.7)	6.3 (±5.0)	NS
**Relative peak EAdi, %**	78.6 (±48.2)	60.3 (±35.5)	52.6 (±16.1)	63.9 (±19.7)	60.8 (±23.5)	64.5 (±23.6)	71.5 (±30.4)	61 (±35.4)	NS
**Pressure transmission to trachea, %**	85.8 (±59.1)	55.7 (±51.3)	69.5 (±43.1)	72.1 (±52.9)	68.9 (±42.8)	72.1 (±43.2)	66.8 (±40.8)	70.2 (±37.9)	NS

Results are presented as mean (± SD). a.u., arbitrary units; ALI, acute lung injury; EAdi, diaphragm electrical activity; NS, not significant.

The measured end-expiratory pressure during the 6 hours of VC started at 4 ± 1.2 cm H_2_O one hour after HCl injury, and was slightly lower at the end of the 6 h (2.7 ± 1.5 cm H_2_O), *P* = 0.100 (Figure 4A). During NIV-NAVA, the applied extrinsic PEEP was zero. Although not significant, there was a tendency for end-expiratory tracheal pressure to increase (1.8 ± 0.8 cm H_2_O at hour 1 to 2.6 ± 1.2 cm H_2_O at hour 6 (Figure 4B). Table 1 summarizes the vital signs for both VC and NIV-NAVA groups throughout the protocol.

During NIV-NAVA, there were no significant changes in the inspiratory EAdi (in absolute values), nor the relative diaphragm activation, during the 6 hours (Table 2). The inspiratory EAdi values during the 6 hours indicated on average, 41% de-activation of the diaphragm at the titrated NAVA level. No significant changes in tonic EAdi were found throughout the 6 hours, although there was a tendency for an increase in hours 5 and 6 (Table 2). Sighs (large neural inspirations) were intermittently observed (range 0 to 9 per hour).

### Lung injury

**Figure 5:**
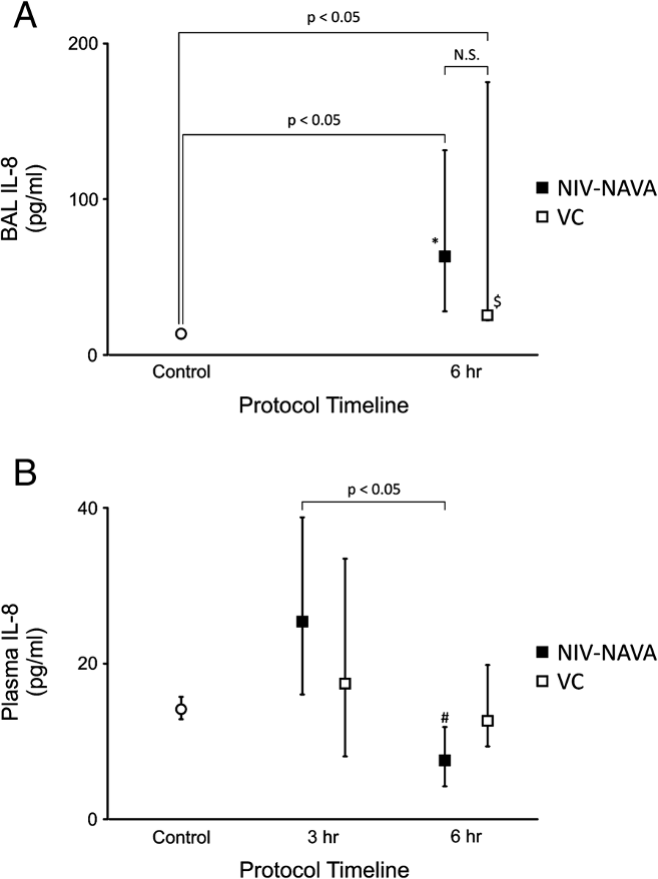
**Markers of lung injury for volume control (VC) and non-invasive ventilation with neurally adjusted ventilator assist (NIV-NAVA). (A)** Values of IL-8 in bronchoalveolar lavage fluid (BAL IL8) were significantly different from control for both VC and NIV-NAVA, but not significantly different from each other. **(B)** Values of plasma IL-8 after 6 hours of NIV-NAVA were significantly lower at 6 hours of NIV-NAVA. Values are median and 25th and 75th interquartile ranges. *NIV-NAVA, significantly different from pre-acute lung injury (Pre-ALI). ^**$**^VC significantly different from Pre-ALI. ^#^NIV-NAVA significantly different from 3 hours.

For the different lung regions, separately, and for the lung as a whole, the mean lung wet-dry ratio was not significantly different between NIV-NAVA (5.3 ± 0.5) and VC (5.3 ± 0.3). Compared to controls, IL8 concentration in the BAL was significantly increased for both the NIV-NAVA and VC groups at the end of the 6 hours, but the groups were not significantly different from each other (Figure 5A). Compared to hour 3 (which was the first post-ALI measurement), plasma IL-8 was significantly lower at hour 6 for the NIV-NAVA group, but not for the VC group (Figure 5B).

**Figure 6:**
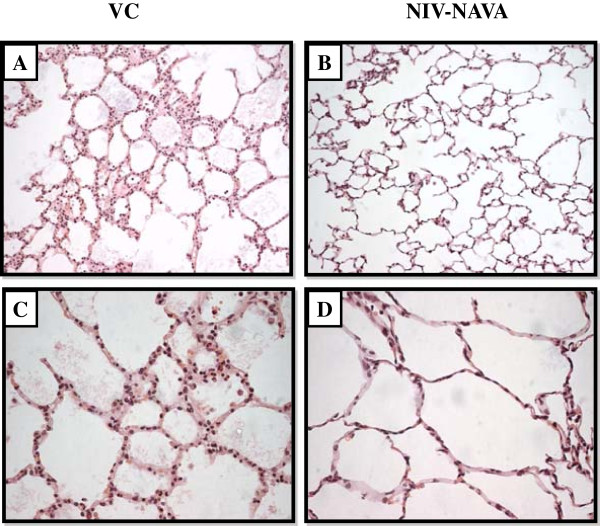
**Representative photomicrographs of lungs stained with hematoxylin-eosin.** Left panels **A** and **C**: animals ventilated with volume-controlled ventilation (VC); right panels **B** and **D**: animals ventilated with non-invasive ventilation with neurally adjusted ventilator assist (NIV-NAVA). **(A and** **B)** Overview of lungs sections at 200× magnification. **(C and** **D)** Close-up of alveoli (400× magnification) showing edema, hemorrhage and neutrophil infiltration.

Figure 6 shows representative photomicrographs of lungs stained with hematoxylin-eosin. Histological analysis showed edema, hemorrhage and neutrophil infiltration at the end of the 6-hour protocol. The severity of lung injury was more pronounced in volume-controlled ventilated animals (left panels) compared to NIV-NAVA ventilated animals (right panels).

**Figure 7:**
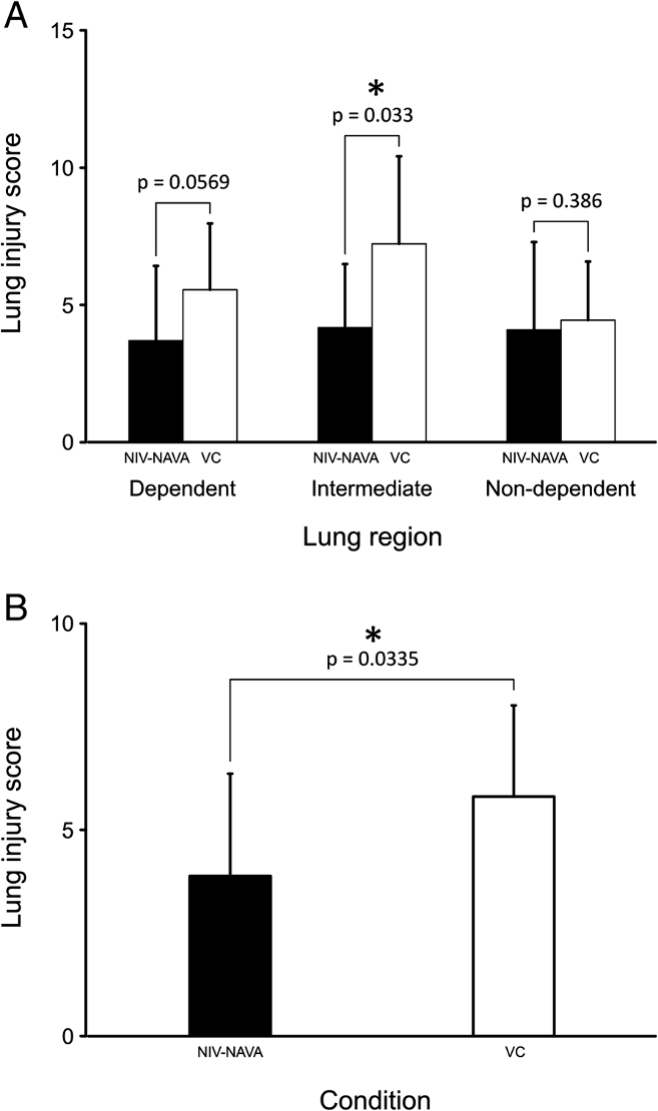
**Lung injury score for volume control (VC) animals and non-invasive ventilation with neurally adjusted ventilator assist (NIV-NVA) animals. (A)** During NIV-NAVA (black bars), the lung injury score for the middle portion of the lung showed significantly lower values than the VC group (white bars). **(B)** Average lung injury score was lower for NIV-NAVA (solid black bar) than the VC arm (white bar). Values are mean ± SD.

The lung injury score (LIS) was significantly lower in the middle (that is, the intermediate/transition) zone of the lung for the NIV-NAVA group, but was not different for the independent and dependent regions (Figure 7A). The LIS averaged over the entire lung was significantly lower for the NIV-NAVA group (*P* = 0.03) (Figure 7B).

## Discussion

The main finding of the present study is that non-invasive ventilation, with a synchronized and adaptable assist, was found to be equally - if not more - protective than a low volume, invasive ventilation mode (volume control with 6 mL/Kg and PEEP). Overall, in the NIV-NAVA arm, we found recovery of dynamic compliance and P/F ratio after 6 hours of ventilation, a lower lung injury score, and a decrease in plasma IL-8.

The two ventilation arms in the present study were clearly different, and it was not possible to match the ventilator patterns. In the VC arm we used the previously reported lung protective strategy as the gold standard [22]. Animals were intubated and paralyzed and all ventilator settings (target Vt, respiratory rate, and PEEP) were imposed and non-adaptable.

In the NIV-NAVA arm, the animals were ventilated non-invasively, and their spontaneous breathing efforts were assisted by a mode of ventilation synchronized and proportional to their neural respiratory drive. With NIV-NAVA therefore, the animals were “free to choose” their own ventilator pressures, and respiratory rates [10]. Non-invasive ventilation could be delivered effectively without glottal closure [23]. The diaphragm remained active not only during inspiration, but partially also during exhalation (so-called tonic EAdi), possibly preventing de-recruitment of the lungs [12].

In addition, because there was a nasal prong to deliver the assist, the upper airways may have been free to participate in regulating end-expiratory volume, by activation of laryngeal and pharyngeal muscles on exhalation, as has been suggested by others [9, 24]. Kosch *et al*. [24] measured diaphragm activity and upper airway muscle activity in children and concluded that “braking mechanisms in infants interact with vagal reflex mechanisms that modulate respiratory cycle timing to influence both the dynamic maintenance of end-expiratory lung volume and ventilation”. Measurement of the electrical activity of the upper airway constrictor muscles (such as the thyroarytenoid or posterior cricoarytenoid muscles) may provide evidence for this in future studies.

With respect to VILI, there is a consensus that the ventilation strategy should avoid overdistension (volutrauma, barotrauma) and cyclic alveolar recruitment and de-recruitment (atelectrauma) [1]. In contrast to the VC arm, no PEEP could be effectively applied during NIV-NAVA because of the leak, and the prototype being used (Servo300) did not have a dedicated NIV mode (note that with today’s commercially available ventilator, there is a dedicated NIV-NAVA mode with leak compensation that is able to deliver adequate PEEP). Despite the fact that no external PEEP was applied, the end-expiratory pressure measured in the trachea at the end of the NIV-NAVA protocol was almost 3 cm H_2_O (Figure 4B). Although we did not measure end-expiratory lung volume per se, our physiological data suggest that the combination of spontaneous breathing (and synchronized ventilation), with the liberated upper airways may have contributed to a reduction in atelectrauma. Several spontaneous breathing strategies may have contributed to this, namely increased respiratory rate (which was higher after lung injury), increased tonic EAdi (which had a tendency to increase at the end of 6 hours), smaller delta pressures generating lower shear forces, and the possible adduction of the vocal cords during expiration (increased expiratory resistance, not measured). Further evidence of sufficient end-expiratory lung volume was that oxygen saturation recovered throughout the 6 hours of NIV-NAVA (Table 1). Given our morphometric data, the main effect of NIV-NAVA in reducing lung injury appears to have occurred in the transition zone between dependent and non-dependent lung zones. This suggests that during NIV-NAVA while the animals breathed spontaneously and in synchrony with the ventilator, without by-passing the upper airways with an endotracheal tube, de-recruitment may have been minimized, leading to protection from atelectrauma. In the VC arm, one could argue that an initial setting of PEEP of 5 cm H2O may have been considered low for experimental lung injury (compare to 8 cm H2O in Brander *et al*. [3]), however the severity of lung injury in that study was greater because two rounds of intratracheal acid installation were performed. After 3 hours of the same protective VC strategy (6 mL/Kg), Brander *et al*. [3] were able to ventilate with a PEEP setting of 5 cm H2O.

Regarding overdistension, several experimental studies have shown downregulation of the EAdi during NAVA, preventing excessive levels of assist [12–14, 25]. It was suggested that this reflex termination of assist and downregulation of EAdi is a vagally mediated reflex sensitive to lung stretch [25]. This prevention of over-assist may also have contributed to the lung protection observed. During non-invasive NAVA, however, it was not possible to measure tidal volume reliably (due to the leak), and therefore, we could not compare Vt relative to the 6 mL/Kg in the VC arm. Therefore, with respect to volutrauma/barotrauma and the role of transpulmonary pressure, the amount of assist delivered during the two types of ventilation should be addressed. The peak ventilator pressures delivered during VC reached about 12 cm H_2_O at hour 5. The peak inspiratory tracheal pressures during NIV-NAVA were in the range of 6 to 7 cm H_2_O. As the animals in the NIV-NAVA arm were spontaneously breathing, their inspiratory effort (not measured) also contributed to the transpulmonary pressure. Based on our previous work in the same animal model [10], the esophageal pressure-swings during inspiration were about -3 cm H_2_O. Summed with the mean expiratory pressure in the trachea of 3 cm H_2_O, the transpulmonary pressure could be estimated to be approximately 12 to 13 cm H_2_O, very similar to the VC arm.

Another approach to estimating how much assist was delivered was by examining the EAdi values. During NIV-NAVA, the NAVA level was determined based on a titration method described previously [13] (at the beginning of the titration procedure, a period of zero NAVA level is used to obtain the highest diaphragm activation). In the present study, the animals were breathing at 60% of their maximum diaphragm activation (in other words they were de-activated, or *unloaded* by 40%).

Throughout the 6 hours of VC ventilation, the peak pressure required to reach 6 mL/Kg increased significantly at hours 5 and 6, indicating worsened respiratory mechanics over time, in accordance with the results of Brander *et al*. [3]. A significant reduction in static compliance was also observed during the 6-hour VC protocol, indicating perhaps a stiffer lung during VC ventilation. It was not possible to measure static compliance during the NIV-NAVA arm of the protocol since the animals were spontaneously breathing. We did, however, re-paralyze the animals at the end of the 6 hours to measure their compliance.

Dynamic compliance recovered in the NIV-NAVA group, perhaps because they were permitted to adopt a spontaneous and variable breathing pattern, which allowed recruitment of the lung both on inspiration and expiration. Sighs were observed intermittently. Coisel *et al*. [26] have suggested that the increased variability in breathing pattern observed with NAVA may lead to improvements in PaO_2_.

Patient-ventilator asynchrony is one of the main reasons non-invasive ventilation fails clinically [27]. In the present study, both ventilation arms were similar in that “fighting of the ventilator” did not occur. In the VC arm, spontaneous breathing activity was not present, whereas during NIV-NAVA, synchrony is achieved, regardless of leaks [10, 28, 29].

The present study has some of limitations. First, in this study, we compared two conditions of mechanical ventilation, where four parameters change: (i) invasive versus non-invasive, (ii) neuromuscular paralysis versus spontaneous breathing, (iii) fixed volume delivery versus variable and proportional assist, and (iv) applied PEEP versus zero PEEP. This actually is the first long-term study (6 hours) using NIV-NAVA under very challenging conditions (hypoxemic respiratory failure, large leak, high respiratory rate, and no capability of applying PEEP). As an initial validation, we chose a direct comparison to a standard experimental lung protective mode (VC). Of course, future studies could aim more specifically at evaluating the individual influences (paralysis, proportionality, synchrony, PEEP, et cetera, on VILI).

Second, one could critique that we did not use a standard mode of NIV, such as NIV-PSV (pressure support ventilation (PSV)), for comparison to NIV-NAVA. Previous work in our laboratory has shown the difficulties of applying NIV-PSV in this model [30]. Because of the large leak, asynchrony can be anticipated during NIV-PSV, adding an additional factor to consider when evaluating VILI. Also, we have demonstrated in conscious animals that the upper airways *fight* the ventilator on inspiration (glottal closure) during NIV-PSV, a phenomena that was not observed during NIV-NAVA [23].

It cannot be neglected that two animals in the NIV-NAVA arm regurgitated. Despite there being no evidence for aspiration in our study (that is, no changes in breathing pattern or P/F ratio), the issue of airway protection and the risk of aspiration should always be considered when applying all modes of NIV (not just NIV-NAVA).

Since this was an experimental study, we do not know if the results of the present study have any relevance to human patients. The use of NIV in patients with ALI and acute respiratory distress syndrome (ARDS) remains controversial, with studies showing a high failure rate of NIV in this group [31]. However, when implemented early, the use of NIV for ALI has been shown to be safe and to improve clinical outcome [32].

Conscious patients undergoing NIV-NAVA may or may not tolerate an EAdi catheter. This certainly depends on the patient’s status, the clinical setting and practice of feeding during NIV, and the interface being used, amongst others. Smaller EAdi catheters (8-F) are commercially available for adult patients undergoing NIV who do not require feeding. In addition, at the moment, the authors do not suggest the clinical use of zero PEEP in humans with hypoxemic respiratory failure during non-invasive ventilation. This needs to be addressed in a future clinical study.

## Conclusions

In experimental animals with ALI, NIV-NAVA is equally as lung-protective, or better, than VC with 6 mL/Kg with PEEP. Spontaneous breathing and upper airway liberation may have contributed to adequate lung protection.

## Key messages

- NAVA is particularly promising for NIV, because it is able to provide synchronized assist and efficiently unload respiratory muscles even in the presence of large air leaks
- In an animal model of HCl aspiration-induced ALI we have shown that NIV-NAVA is at least as protective as the standard lung-protective invasive ventilation mode (VC ventilation with PEEP and low Vt)
- The combination of (i) freedom to choose breathing pattern, (ii) liberated upper airways, (iii) integration with vagally mediated lung-protective reflexes may have contributed to the adequate lung protection observed during NIV-NAVA in this experimental model.

## Electronic supplementary material

Additional file 1: **Materials and methods (details).** This is a file with additional details about the material and methods. (DOCX 29 KB)

## Abbreviations

ALI: acute lung injury
BAL: bronchoalveolar lavage
Cdyn: dynamic compliance
EAdi: electrical activity of the diaphragm
ELISA: enzyme-linked immunosorbent assay
FIO2: inspired oxygen fraction
HCl: hydrochloric acid
IL: interleukin
LIS: lung injury score
NAVA: neurally adjusted ventilatory assist
NIV: non invasive ventilation
PaO2: partial arterial pressure of oxygen
PEEP: positive end-expiratory pressure
Pplat: plateau airway pressure
Ptr: tracheal pressure
Pvent: ventilator pressure
VC: volume-control
VILI: ventilator-induced lung injury
Vt: tidal volume.
