# Supplementary material for: Lung protection during non-invasive synchronized assist versus volume control in rabbits
Source: Crit Care. 2014 Jan 23;18(1):R22. doi: 10.1186/cc13706 (PMC4057206; doi:10.1186/cc13706)
Supplement: Supplementary file 1 — Additional file 1: Materials and methods (details). This is a file with additional details about the material and methods. (DOCX 29 KB) [file 13054_2013_2859_MOESM1_ESM.docx]

**Additional File 1:**

# Lung Injury during Non-invasive Synchronized Assist versus Volume Control in Rabbits

##### Lucia Mirabella^1^MD, Giacomo Grasselli^2^MD, Jack J. Haitsma^3^MD, Haibo Zhang^3,4^ MD, Arthur S. Slutsky^3,4^MD , Christer Sinderby^3, 4^ PhD, *Jennifer Beck ^3,5^PhD

^1^Department of Anesthesiology, University of Foggia, viale Pinto 1, 71122 Foggia (FG), Italy

^2^Department of Experimental Medicine, University of Milano-Bicocca, via Cadore 48, 20900 Monza (MB), Italy

^3^Keenan Research Centre for Biomedical Science of St. Michael’s Hospital; Department of Critical Care, St. Michael's Hospital, 30 Bond Street, Toronto, Ontario, Canada, M5B1W8

^4^Department of Medicine and Interdepartmental Division of Critical Care Medicine, University of Toronto, Toronto, Canada

^5^Department of Pediatrics, University of Toronto, Toronto, Canada

**MATERIALS AND METHODS**

Details:

The study was approved by St.Michael’s Hospital Animal Care and Use Committee. Care and handling of the animals was in accordance with the Canadian Council on Animal Care.

*Animal preparation*

Twenty male New Zealand white rabbits with a mean body weight of 3.1 ± 0.4 Kg were studied for the ventilation protocol. Five additional animals were included to obtain “healthy/unventilated” control values for bronchoalveolar lavage (BAL) and plasma interleukin-8 (IL-8). These animals were not lung-injured and were not ventilated. Only control values were obtained.

All the ventilated animals (n=20) were tracheostomized, and an endotracheal tube (4.0 ID) was installed into the trachea but below the larynx **(Figure 1, main manuscript)**. A ligature was placed around the trachea and tube to minimize the leak. The ventilator used was a Servo300 ventilator (Maquet Critical Care, Solna, Sweden). In the VC arm, the ventilator was connected to the endotracheal tube (**see Figure 1, main manuscript, VC setup**) at the tracheostomy. In the NIV-NAVA arm, the endotracheal tube (4.0 ID but cut at both ends with a side port) was inserted both proximally and distally into the trachea. The ventilator was connected to a single nasal prong (2.5 mm OD, placed 2 cm into one nostril), as previously described (E1). **(Figure 1, main manuscript, NIV-NAVA setup)**. Both ventilation groups had the same physical placement of a tube in the trachea.

All animals received continuous intravenous infusion of ketamine hydrocloride (40 mg/Kg/h), xylazine (4 mg/Kg/h) and lactated Ringer’s solution (5 mL/Kg/h). With this anesthetic regime, animals are breathing spontaneously (E1-E4). Arterial blood pressure, transcutaneous oxygen saturation, body temperature, and heart rate were monitored throughout the study.

Pressure measurements:

In VC, ventilator-delivered pressure (Pvent) was measured at the Y-piece connected to the endotracheal tube (VC setup).

During NIV-NAVA, ventilator-delivered pressure was measured at the nasal prong (Pprong) and Ptr was measured via a side-port of the endotracheal tube **(Figure 1, main manuscript)**.

As described previously, an 8-Fr catheter equipped with electrodes to measure EAdi was positioned in the esophagus (E1, E2). The animals were in supine position, with slight elevation of the head.

# *Experimental Protocol* (Figure 2, main manuscript)

After instrumentation, all the animals were ventilated for 15-20 minutes before induction of acute lung injury (“Pre ALI”) with either volume control (VC) mode (see below) or NIV-NAVA and then for 6 hours after lung injury with the same mode.

VC (*paralyzed*): Ten rabbits were ventilated in volume control (VC) before and after lung injury (Vt 6 mL/Kg; initial Peep 5 cmH_2_O) and received continuous infusion of pancuronium (0.1-0.2 mg/Kg/h). Ventilator rate was imposed by the investigator and adjusted throughout the study based on the previous hour’s blood gases. FIO2 was set to 50%.

NIV-NAVA (*spontaneous breathing*): Ten rabbits were ventilated with NIV-NAVA before and after induction of ALI. The method for NAVA has been previously described in detail (E1-E4). Briefly, during NAVA, the processed EAdi waveform controls the timing (trigger and cycle-off) and the level of assist. The amount of assist is adjusted by the NAVA level, a proportionality constant between EAdi and pressure delivered. Ventilator rate is spontaneously chosen by the animal.

The NAVA level was titrated (E4-E7) before and after ALI. This involves a stepwise increase in the NAVA level (starting from zero cm H2O per a.u. of EAdi, where a maximum diaphragm activation can be measured) every 5-6 breaths, until a plateau is observed in the ventilator pressure trend; the NAVA level was set according to this plateau (E4).

During NIV-NAVA, no effective PEEP could be applied because of the leak (Servo 300 ventilator does not have dedicated NIV mode). FIO2 was maintained at 50%.

*Acute lung injury procedure*

The VC animals were already paralyzed for Pre ALI measurements. For the NIV-NAVA group, after Pre ALI measurements, animals received repeated boluses of pancuronium (0.05 mg intravenously) until the EAdi disappeared and VC ventilation was implemented (Vt 6 mL/Kg; Peep 5 cmH_2_O; FiO_2_ 50%). During neuromuscular paralysis (before and after ALI), the animals in the NIV-NAVA arm were ventilated at the tracheotomy in the VC mode.

For both the VC groups and NIV-NAVA, total respiratory dynamic compliance (Cdyn) was measured during the paralysis (VC mode) before ALI with the formula: Vt/(Peak-PEEP) and units ml/cmH2O.

Then, induction of lung injury was performed by intratracheal instillation of hydrochloric acid (pH 1.5) (1.5 mL/Kg) followed by a recruitment maneuver (continuous positive airway pressure of 25 cmH_2_O for about 5 seconds) as previously described (E1, E2).

The Cdyn measurement was repeated after induction of lung injury with the same VC settings as before injury.

In both groups the animals were ventilated for six hours (paralyzed for VC, spontaneous breathing for NIV-NAVA), and arterial blood samples were taken before ALI, 5 minutes after induction of ALI (Post ALI), and hourly thereafter. All other measurements (EAdi, Pvent, Pprong, Ptr) were recorded for the last 20 minutes of each hour. At the end of the protocol, Cdyn was re-measured in both groups using the same ventilator settings as before; animals in the NIV-NAVA arm were re-paralyzed immediately prior to these measurements.

*Evaluation of lung injury at end of 6 hours*

After sacrificing the animals with an overdose of Xylazine and Ketamine, the heart-lung block was removed, the lungs were inflated and the main left side bronchus and the right lower lobe bronchus were tightly occluded. Twenty mL of normal saline was instilled intratracheally and filled the unoccluded right lung for bronchoalveolar lavage (BAL). It was then aspirated and immediately centrifuged at 4°C at 3000 rpm and the supernatant stored in aliquots at -80°C for later analysis.

Lung wet-to-dry ratio was measured in different the parts of the right lower lobe, namely, dependent, intermediate, and non-dependent (cut and weighed before and after exposure to 40°C for 96 hours) [(lung wet weight- lung dry weight)/lung dry weight]. The remaining right lung was snap frozen in liquid nitrogen and stored in -80°C for later analysis of tissue IL-8.

Rabbit IL-8 was measured in BAL fluid and plasma (hours 3 and 6) using a human IL-8 ELISA kit (Biosource International Camarillo, CA) since the anti-human IL-8 antibody in the kit cross-reacts with rabbit IL-8 (E2).

In general, the bioassays for rabbits are limited to a few, and IL-8 is probably the best one available to represent inflammation in rabbits. Ware et al (E8) state that "The best performing biomarkers were the neutrophil chemotactic factor, IL-8, and SP-D, a product of alveolar type 2 cells, supporting the concept that acute inflammation and alveolar epithelial injury are important pathogenetic pathways in human ALI/ARDS." (E8, E9).

*Histology*

The left lung was taken for histopathology and fixed in 4% paraformaldehyde. The lung was sectioned in dependent, intermediate and non-dependent tissue blocks and embedded in paraffin, and stained with hematoxylin and eosin.

The analyzing pathologist was not informed about the study purpose, blinded for the samples and only asked to score for lung injury. Scoring was done on: alveolar distension, alveolar collapse, perivascular haemorrhage, alveolar haemorrhage, perivascular edema, infiltration by polymorphonuclear leukocytes, alveolar membranes, intraalveolar edema in a range of none, mild, moderate and severe (score 0, 1, 2, or 3, respectively) for descriptive purposes as previously described (E2).

# *Analysis of Ventilatory Variables*

Analysis of EAdi and respiratory variables was performed offline using custom made software (Neurovent Research Inc, Toronto, Canada) as previously described [E1-E4]. Peak Pvent and Ptr (in the NIV-NAVA arm) were characterized by the highest pressure deflection per breath. Mean tracheal pressure during expiration was also measured. EAdi was analyzed for the phasic (inspiratory deflection) and tonic (end-exhalation EAdi) activities (E10).

For both NIV-NAVA and VC, the average value of each variable was calculated for the last 2 minutes of the recorded periods.

# *Statistics*

Data are presented as mean ± SD or median (quartiles) where appropriate (depending on normal distribution). Two way repeated measures ANOVA was used to compare the parameters in the measured variables over time and between modes. Post-hoc analysis was performed using the Student Newman-Keuls Test.

T-tests were used to compare the IL-8 concentration in blood samples and BAL, as well as for the LIS. For all statistical tests, a significant difference was defined as p<0. 05. SigmaStat was used for statistical analyses (Sigmastat, Jandel Scientific, San Rafael, CA).

**REFERENCES**

E1. Beck J, Brander L, Slutsky AS, Reilly MC, Dunn MS, Sinderby C: [**Non-invasive neurally adjusted ventilatory assist in rabbits with acute lung injury**.](http://www.ncbi.nlm.nih.gov/pubmed/17960364) *Intensive Care Med* 2008,**34**:316-323

E2. Brander L, Sinderby C, Lecomte F, Leong-Poi H, Bell D, Beck J, Tsoporis JN, Vaschetto R, Schultz MJ, Parker TG, Villar J, Zhang H, Slutsky AS: **Neurally adjusted ventilatory assist decreases ventilator-induced lung injury and non-pulmonary organ dysfunction in rabbits with acute lung injury**. *Intensive Care Med* 2009,**35**:1979-1989

E3. Allo JC, Beck JC, Brander L, Brunet F, Slutsky AS, Sinderby CA: **Influence of neurally adjusted ventilatory assist and positive end-expiratory pressure on breathing pattern in rabbits with acute lung injury**. *Crit Care Med* 2006,**34**:2997-3004

E4. Lecomte F, Brander L, Jalde F, Beck J, Qui H, Elie C, Slutsky AS, Brunet F, Sinderby C: **Physiological response to increasing levels of neurally adjusted ventilatory assist (NAVA)**. *Respir Physiol Neurobiol* 2009,**166**:117-124

E5. Brander L, Leong-Poi H, Beck J, Brunet F, Hutchison SJ, Slutsky AS, Sinderby C: **Titration and implementation of neurally adjusted ventilatory assist in critically ill patients**. *Chest* 2009,**135**:695-703

E6. Passath C, Takala J, Tuchscherer D, Jakob SM, Sinderby C, Brander L: **Physiologic response to changing positive end-expiratory pressure during neurally adjusted ventilatory assist in sedated, critically ill adults**. *Chest* 2010,**138**:578-587

E7. Tuchscherer D, Z'graggen WJ, Passath C, Takala J, Sinderby C, Brander L: **Neurally adjusted ventilatory assist in patients with critical illness-associated polyneuromyopathy**. *Intensive Care Med* 2011,**37**:1951-1961

E8. Ware LB, Koyama T, Billheimer DD, Wu W, Bernard GR, Thompson BT, Brower RG, Standiford TJ, Martin TR, Matthay MA; NHLBI ARDS Clinical Trials Network: [**Prognostic and pathogenetic value of combining clinical and biochemical indices in patients with acute lung injury**.](http://www.ncbi.nlm.nih.gov/pubmed/19858233) *Chest.* 2010, **137**:288-296

E9. Papadopoulou C, Corrigall V, Taylor PR, Poston RN: [**The role of the chemokines MCP-1, GRO-alpha, IL-8 and their receptors in the adhesion of monocytic cells to human atherosclerotic plaques**.](http://www.ncbi.nlm.nih.gov/pubmed/18579408) *Cytokine*. 2008,43:181-186.

E10. Beck J, Reilly M, Grasselli G, Qui H, Slutsky AS, Dunn MS, Sinderby CA: **Characterization of neural breathing pattern in spontaneously breathing preterm infants**. *Pediatr Res* 2011,**70**:607-613
